# Structural Remodeling of the Extracellular Matrix in Arteriogenesis: A Review

**DOI:** 10.3389/fcvm.2021.761007

**Published:** 2021-11-05

**Authors:** Rohan Kulkarni, Elizabeth Andraska, Ryan McEnaney

**Affiliations:** ^1^Division of Vascular Surgery, University of Pittsburgh Medical Center, Pittsburgh, PA, United States; ^2^Veterans Affairs Hospitals Pittsburgh Healthcare System, Pittsburgh, PA, United States

**Keywords:** arteriogenesis, extracellular matrix, elastic fiber, outward remodeling, collateral arteries, arterial occlusive disease

## Abstract

Lower extremity arterial occlusive disease (AOD) results in significant morbidity and mortality for the population, with up to 10% of patients ultimately requiring amputation. An alternative method for non-surgical revascularization which is yet to be fully understood is the optimization of the body's own natural collateral arterial network in a process known as arteriogenesis. Under conditions of conductance vessel stenosis or occlusion resulting in increased flow, shear forces, and pressure gradients within collaterals, positive remodeling occurs to increase the diameter and capacity of these vessels. The creation of a distal arteriovenous fistula (AVF) will drive increased arteriogenesis as compared to collateral formation with the occlusion of a conductance vessel alone by further increasing flow through these arterioles, demonstrating the capacity for arteriogenesis to form larger, more efficient collaterals beyond what is spontaneously achieved after arterial occlusion. Arteries rely on an extracellular matrix (ECM) composed of elastic fibers and collagens that provide stability under hemodynamic stress, and ECM remodeling is necessary to allow for increased diameter and flow conductance in mature arterial structures. When positive remodeling occurs, digestion of lamella and the internal elastic lamina (IEL) by matrix metalloproteinases (MMPs) and other elastases results in the rearrangement and thinning of elastic structures and may be replaced with disordered elastin synthesis without recovery of elastic function. This results in transmission of wall strain to collagen and potential for aneurysmal degeneration along collateral networks, as is seen in the pancreaticoduodenal artery (PDA) after celiac occlusion and inferior mesenteric artery (IMA) with concurrent celiac and superior mesenteric artery (SMA) occlusions. Further understanding into the development of collaterals is required to both better understand aneurysmal degeneration and optimize collateral formation in AOD.

## Introduction

The incidence of lower extremity arterial occlusive disease (AOD) has continued to increase over the past several decades resulting in significant morbidity and mortality for the population. Symptoms progress slowly after onset, however between 5 and 10 years after diagnosis 20–30% of patients will experience progressive symptoms requiring intervention with up to 10% requiring amputation (1, 2). Non-surgical therapies for symptomatic patients include behavioral and pharmacological risk factor modification and exercise therapy. Revascularization, however, depends on invasive interventions like endoluminal angioplasty and stenting or surgical bypass, as examples. Despite improving methods and technologies, revascularization procedures pose some risk to the individual and have anatomical requirements. As a result, some patients are not suitable candidates for revascularization.

**Figure 1:**
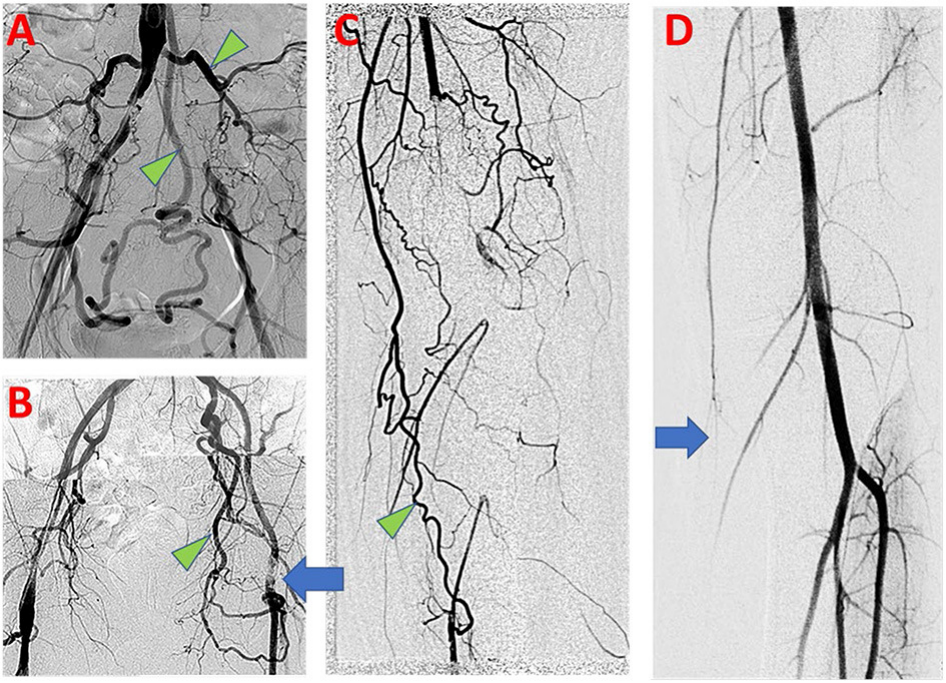
Clinical findings of PAD in symptomatic patients. **(A)** Aortoiliac arteriogram demonstrates bilateral common iliac artery occlusive disease, with evidence of compensation by large lumbar and inferior mesenteric arterial collaterals (Green arrowhead). **(B)** Left common femoral artery occlusive disease (blue arrow) with prominent developed left obturator artery collateral (Green arrowhead). **(C)** Left lower limb popliteal artery occlusion with numerous collateral arteries, including developed branches of the descending genicular artery (Green arrowhead). **(D)** Normal left lower limb arteriogram shows small size and limited opacification of branches of the descending genicular artery at baseline (Blue arrow).

Fortunately, individuals with AOD often benefit from some level of natural adaptation which manifests as development of collateral arterial networks. When large conductance arteries become obstructed, flow patterns immediately change and distal perfusion becomes increasingly dependent on collateral development (3). This process is known as arteriogenesis and involves the outward remodeling and growth of pre-existing arterioles to create an effective collateral network (4). Compared with angiogenesis, which results in the local growth and development of *de novo* capillaries in ischemic beds, arteriogenesis is the primary means by which blood flow is recovered to distal tissue (5). Effective, functional collateral arteries may minimize clinical symptoms of AOD and allow for conservative management of symptoms (6, 7). Functional coronary collateral networks associate with reduced mortality as well (3, 8–10). Collateral artery networks can readily be identified on arteriograms obtained from patients with peripheral AOD as demonstrated in Figure 1.

Unfortunately, collateral vessels formed do not spontaneously restore maximal conductance to levels equal to that of the occluded artery they seek to replace. In an early experimental model of arterial occlusion, spontaneously developing collaterals only restore 35–40% of maximum conductance of the occluded artery (11) and does not exceed 50% (12). We have previously shown, however, that collateral capacity has a significantly higher ceiling than what may be achieved spontaneously after large artery occlusion, suggesting potential utility for pharmacotherapies that may augment collateral network development (13). In the following review, we describe current knowledge of mechanisms of arteriogenesis.

### Origins of Collateral Arteries

Collateral arteries naturally develop from pre-existing arterial connections across arterial territories that span an occluded conductance vessel (14). Although not readily identifiable by conventional arteriogram in the absence of pathology (Figure 1D), collateral anastomoses are widely present among healthy individuals and persist from development (15). Such arterial anastomoses have been shown to exist in all arterial territories, but may vary by tissue and species, leading to differences in perfusion protection in the case of arterial occlusion (16). Among mice strains, the differences in baseline collateral connections have been shown to be genetically determined (17–19). In human coronary disease patients, Hollander et al. showed that improved human coronary collateral flow indices was associated with higher palmar collateral flow indices, indicating coherence of collateral connections between different circulatory beds within an individual (20).

By Longland's classification, collateral arterial pathways are comprised of three components; the proximal, communicating, and the distal branches, more conveniently referred to as the stem, the midzone, and the re-entrant arteries (21). The midzone is the area of greatest focus because this is where the greatest degree of outward structural remodeling occurs, effectively transforming resistance vessels such as arterioles into collateral arteries which have a role in blood flow conductance.

### Arterial Extracellular Matrix Structure

The arterial extracellular matrix (ECM) is primarily composed of elastic fibers and various collagens and is necessary to provide structural stability to the vessel under conditions of hemodynamic stress. Elastin stores energy and distributes stress along the vessel wall while collagen, which is primarily located in the adventitia, prevents over distension of the vessel and provides a resilient framework, though it is unable to store energy (22). In the setting of excessive wall strain, such as what occurs in mid-zone collaterals during arterial occlusive disease, collagen in the adventitia limits increases in arterial diameter and over-stretching of elastic tissues. This is evidenced in the straightening of collagen fibers and is known as the “two-phase” material property which supports arterial function and stability (23–27). Unfortunately, this sets limits on potential outward diameter expansion and must be overcome for collateral enlargement to occur.

Elastin fibers consist of a heavily crosslinked, dense elastin protein core surrounded by peripherally oriented proteins and proteoglycans. These fibers have an estimated half-life approaching the human lifespan and are created predominantly in neonatal and early postnatal life with very little new elastin made during adulthood (28, 29). Their construction consists of tropopelastin monomers produced by vascular smooth muscle cells (VSMCs) and endothelial cells which then self-assemble in the extracellular space, assisted by a microfibril scaffold. Lysine residues within tropoelastin are modified by the enzyme lysyl oxidase (LOX) which leads to covalent cross-link formation, greatly contributing to the elastic polymer's resilience and durability (28–30). When damaged physiologically or pathologically, elastic fibers can be salvaged if integrity is preserved. Lost elastic fiber integrity may be replaced with disordered elastin synthesis without recovery of elastic function (31–35).

Elastic fibers are present throughout the arterial wall but are most prominently featured in the dense sheets separating rows of resident VSMCs known as lamellae. These lamellae are present within the tunica media. Between the tunica intima and media, a prominent lamina underpins the endothelium and is better known as the internal elastic lamina (IEL). These elastic lamellae contain fenestrations which vary in size and frequency depending on the arterial branch order, and allow for cellular communication, diffusion, and molecular transport (36–39). More importantly, these lamellae become active sites of arterial remodeling during postnatal growth of arterial structures and arteriogenesis (40, 41). Large elastic arteries (such as the common femoral artery) consist of thick, wrinkled elastin with small and rounded fenestrations, while secondary and more distal vessels have a fine meshwork of fibers in place of an established IEL (39). Under low pressure the IEL appears wrinkled and wavy due to redundancy and is observed to flatten at higher pressures as the artery distends.

In small arteries Type IV collagen forms the basement membrane while type I collagen bundles are abundant in the adventitia. Collagen fibers have a similar wavy appearance in all vessels. This baseline variability in ECM structure is significant in that it may have clinical consequences regarding remodeling capacity of these vessels. Alternatively, collagen fibers have half-lives as low as 2 weeks under experimental conditions of hypertension, and likely must be continually synthesized and replaced (42, 43).

### Flow Patterns Regulate Vessel Diameter

As arterial occlusive disease progresses, flow patterns automatically adapt as blood flow follows the path of least resistance. This may result in large flow rate increases among inter-territorial connections. The endothelial cells are influenced by the resulting shear stress elevations imparted by this increased flow and may become activated (5, 44). Arteriogenesis is initiated in response to sustained elevations in shear force gradients (45). Through a mechanism that is not well-elucidated, vascular endothelial cells transduce the increased shear forces and initially respond with endothelial nitric oxide (NO) gene expression as well as cytokine and adhesion molecule release (46).

Assuming laminar conditions, the primary forces acting on the vascular wall are fluid shear stress (FSS) and circumferential wall stress (CWS). FSS is experienced as the frictional force of blood exerted against the vascular wall, specifically the endothelium, which is believed to act as the primary modulator of this input (47). The force of increased flow as interpreted by the endothelium is widely regarded as the initial event leading to vasodilation and downstream chronic vascular remodeling (48). The initial abrupt elevation in shear force followed by a gradual normalization is subsequently associated with remodeling of all three layers of the vascular wall, extracellular matrix, and ultimately yields vessel diameter expansion (49, 50).

Increased volume flow through a vessel has been shown to induce outward remodeling and diameter growth (51–53). As luminal diameter increases, fluid shear stress necessarily drops precipitously, and may provide a “set point” for growth. This self-regulating mechanism, which has been described as the shear stress “set point theory,” states that fluid shear stress at the endothelial level essentially will return to normal, signaling resolution of remodeling. Increases in the vessel radius leads to decreases in wall shear stress and provides the system with a negative feedback autoregulatory loop (48). The mechanism for this process is incompletely understood, however there are theories related to epigenetics and DNA hypermethylation which could influence mechanosensitivity, as well as influences from VEGFR3 in setting a vessels innate setpoint (54, 55).

### Events of Arteriogenesis

#### Increased FSS, Endothelial Mechanotransduction, Vasodilation

Loss of a conductance artery necessarily contributes to altered pathways for blood flow due to shifting in pressure gradients. Pre-existing collateral connections between territories separated by a conductance artery occlusion will be subjected to increased flow and shear stress. The endothelium within these collateral pathways detects the shear alterations through mechanotransduction cascades that are complex and incompletely understood. The endothelial glycocalyx has been shown to be important in endothelial mechanotransduction and its absence leads to diminished arteriogenesis (56, 57). Nucleotides are released extracellularly in response to shear stress and subsequent purinergic receptor activation leads to endothelial cell mediated vasodilation (58–60). Caveolae are sites of signaling activity in response to FSS alterations in cultured endothelial cells and are necessary for flow-mediated remodeling responses (61, 62). Various integrins are implicated in shear force transduction and endothelial-mediated vasodilatory response (63, 64), and the functional remodeling required for arteriogenesis including production of elastase (64–67).

Nitric oxide has been implicated in arteriogenesis, but its role is complex. Endothelia respond to increased shear stress with increased endothelial nitric oxide synthase (eNOS) expression and subsequent production of NO, a potent vasodilator (68, 69). Purinergic receptors activated in response to elevated FSS and are necessary for flow-mediated NO production and subsequent vasodilation (59, 60, 70). Some described arteriogenesis experiments have demonstrated that early perfusion recovery depends on vasodilatory mechanisms, and NO production (71). Additionally, while loss of eNOS alone does not alter arteriogenesis, loss of inducible nitric oxide synthase (iNOS) does inhibit collateral development (72). NO has a role in remodeling via MMP activation, andinhibition of NOS results in a significant decrease in MMP activity (73). Chronic inhibition of NOS reduces diameter enlargement relative to controls and impairs vessel autoregulation to its shear stress set point (74). Notably, loss of endothelium decreases vessel response to chronic flow alterations (75).

Although short-lived vasoactive signals may produce immediate vasoconstriction or vasodilation mediated by VSMC shortening or lengthening, cessation of the signal results in return to baseline vessel diameter. Sustained signal, however, produces additional adaptation of resident VSMCs through “length autoregulation,” reorienting cell-cell and cell-ECM adhesion and thus increasing or decreasing VSMC overlap (76). Such adaptations result in maximal allowable diameter increase of the arterial segment without breakdown of the ECM, referred to as mechanoadaptation (76, 77).

#### Endothelial Expression of Adhesion Molecules, Chemokines, and Leukocyte Recruitment

Endothelial cells activated by prolonged elevations in shear stress begin to express increased levels of vascular cell adhesion molecule-1 (VCAM-1) and intercellular adhesion molecule 1 (ICAM-1). Adhesion molecule expression recruits circulating leukocytes to the developing collateral artery by promoting adherence and transmigration into the developing vessel wall (78–81). Inhibition of ICAM-1 via monoclonal antibodies directly reduces leukocyte migration, and ICAM-1 deficiency reduces collateral perfusion in response to arterial occlusion (82, 83). Endothelial VCAM-1 and ICAM-1 expression in response to flow alterations is regulated in part by thy P2Y_2_ purinergic receptor (84–86), and its absence results in reduced inflammatory cell recruitment and diminished collateral development (87).

Shear stress induced endothelial activation also promotes the release of several cytokines including monocyte chemoattractant protein-1 (MCP-1), TNF-α, and granulocyte macrophage colony stimulating factor (GM-CSF) which importantly attract monocytes (46, 88, 89). MCP-1 attracts monocytes to areas of active remodeling as well as upregulation of cellular adhesion molecule ICAM-1 (79). Local infusions of MCP-1 to developing collateral arteries have been shown to improve arteriogenesis. However, in an experimental model of arteriogenesis it was found that local tissue macrophage proliferation in response to MCP-1 was more important than blood-borne monocyte recruitment for mediating collateral development (90).

Adhesion molecule expression by activated endothelium and local cytokine production recruits a broad population of inflammatory cells to participate in arteriogenesis. Neutrophils appear to be recruited first, and produce cytokines such as midkine, which has been suggested to mediate VEGF release (91, 92). We have also shown that within the first 48 h, there is a significant upregulation of neutrophil elastase transcription in whole vessel analysis, suggesting that neutrophil presence may be important in initiation of ECM remodeling. Neutrophils have a short time presence outside of the circulation, however, and are generally absent later in developing collaterals, suggesting their role may be limited. Natural killer cells and CD4+ T cells have been implicated in arteriogenesis as well (93, 94).

Additionally, administration of lipopolysaccharide (LPS) stimulates TNF-a and is also capable of increasing perivascular concentrations of monocytes/macrophages leading to collateral growth and development (95). Once activated, monocytes, as well as T lymphocytes, release MMPs along with TNF-a and growth factors such as b-FGF and PDGF which serve to induce smooth muscle cell phenotype switching from a contractile to a proliferative phenotype, permitting migration (95–98). It has been shown that intra-arterial injection of MCP-1 and GM-CSF are capable of increasing collateral diameter due to recruitment of circulating monocytes, and is inhibited by monocyte depletion (82, 99, 100). Macrophages have been demonstrated as being important mediators of arteriogenesis (101). Of course, macrophages have complex functions depending on their phenotypes, and as a result may play pro-inflammatory or reparative roles. Even in the absence of flow induced changes, macrophages have been shown to promote degradation of the IEL, a key occurrence to allow for outward remodeling (78, 101, 102).

#### Proliferation of Resident Cells

Collateral arterial growth occurs with the proliferation of resident tissue cells, which can expand the vessel mass nearly 25 times the original (103). Activated endothelium release mitogenic factors as well as promote local proliferation and ECM remodeling. Endothelial proliferation has been shown to precede that of VSMCs and may be directly induced by shear stress activation (95). VSMCs are not exposed to shear forces, and direct control of the phenotypic modulation and proliferation necessary are less well-defined. Typically, VSMCs are maintained in what is known as a contractile phenotype (oriented circumferentially around a vessel) which is typically the differentiated, quiescent form for VSMCs. These cells are separated from the intima by the IEL and contained within their local microenvironment by a basal lamina which envelopes these cells (104). VSMCs will dedifferentiate, reverting to a synthetic phenotype capable of proliferation and migration during the expansion of arteriogenesis (105). FGF and PDGF have been found to be important growth factors involved in upregulating vascular smooth muscle cell (VSMC) growth and differentiation leading to phenotype switching, actin polymerization, and maturation (106–108). Increased PDGF resulting from increased shear is suggested to be an important early factor involved in the cellular adaptation of vessels to flow mediated via the endothelium (109).

As VSMC populations migrate and expand, a neointima forms in the developing collateral artery, appearing as early as 3 days following occlusion (79). Formation of the neointima depends on ECM modifications to remove barriers to migration. VSMCs may release MMPs and plasmin activators (which convert the pro-enzyme plasminogen to active plasmin) and degrade several ECM components (110). Experimental evidence has suggested that VSMC migration and proliferation depend on MMP activity and IEL degradation (111). Unlike instances of neointima formation in intimal hyperplasia or atherosclerosis, in arteriogenesis the increased wall thickness is balanced by overall increase in luminal diameter. Eventually luminal diameter increases enough to reduce local FSS back to within an acceptable range, the mitogenic stimuli dissipate, and VSMCs return to a contractile phenotype.

#### ECM Remodeling—Alteration in Vascular Structure

**Figure 2:**
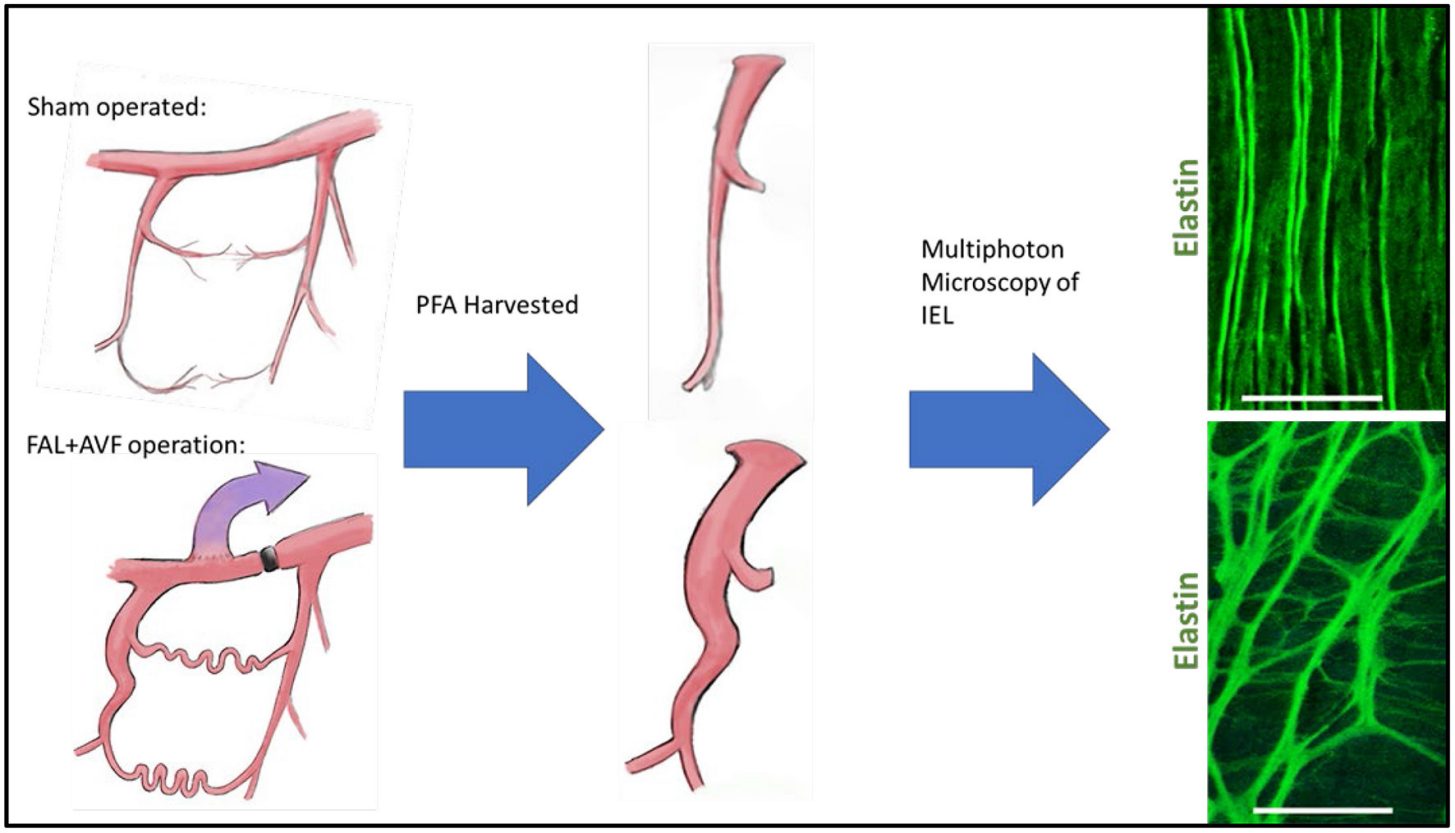
Internal elastic lamina (IEL) remodeling in a rodent model of arteriogenesis. Rat hindlimbs were rendered ischemic by placement of femoral artery ligation with distal arteriovenous fistula or treated as controls with sham operation (incision and exposure without further arterial manipulation) as we have previously described (13). Profunda femoral arteries were harvested at 12 weeks and imaged with multiphoton microscopy. Note the wrinkled topology of the IEL with small regular fenestrations in the control vessel, which is lost in the remodeled vessel as the fenestrations are greatly enlarged. Images acquired with Olympus FV1000MPE utilizing 830 nm laser. Bar = 50 um. Multiphoton microscopy methods described previously (39).

Diameter increase along with ECM remodeling requires the rearrangement of elastic tissue in the internal elastic lamina. Ultimately, elastolysis allows for vessel diameter enlargement, and ECM remodeling is necessary to allow for increased diameter and flow conductance in mature arterial structures (112). In the developing collateral artery, this appears as fenestration enlargement of the IEL, such that outward remodeling during collateral development is achieved while simultaneously maintaining IEL continuity. Others have demonstrated that the fenestrations are the active sites of both outward and inward remodeling of the IEL (40, 41, 113, 114). We have found that after femoral artery ligation with distal arteriovenous fistula creation (FAL + AVF), an initially dense elastin network transforms into a loose meshwork with the general pattern of IEL reorganization, demonstrating increases in fenestration size bordered by cords of branching elastic fibers (Figure 2). Increases in circulating desmosine (an elastin breakdown product) within 1 week after FAL + AVF were found which disappear after 2 weeks, (Andraska et al. submitted to the current issue) supporting that elastin degradation is important early in arteriogenesis. It has been shown that collateral artery development requires activated MMP-2 and MMP-9, and no fragmentation of the IEL noted when administered MMP inhibitors (115). Cathespins, also involved in vascular remodeling, have elastolytic properties as well and are found to be upregulated in developing collateral arteries (116–119).

The damage to the elastic fibers during arteriogenesis must be limited in order to maintain fiber integrity and prevent loss of IEL continuity. Tissue inhibitor of metalloproteinases (TIMP1) and plasminogen activator inhibitor 1 (PAI-1) also play vital roles in collateral development by inhibiting MMP function and preventing excessive breakdown of ECM (111). It would seem that elastic fiber preservation and repair is necessary during arteriogenesis, given that construction of new elastic fibers construction is unlikely to occur.

Cross linking of elastin and collagen fibers is mediated via the enzyme lysyl oxidase (LOX) and is essential to maintaining the integrity of the ECM. LOX expression is increased in remodeling of FAL + AVF arterioles and we found that inhibiting LOX [using β-aminopropionitrile (BAPN)] resulted in rapid fragmentation and loss of continuity of the IEL from collateral arteries. This suggests the role of LOX in the repair and stabilization of proteolytically digested elastic fibers during arteriole remodeling. This suggests the proteolytic balance between breakdown and repair during remodeling, possibly related to cross linking of newly synthesized tropoelastin monomers in the later stages of arteriogenesis (31–34). Notably, increased tropoelastin expression has been seen in models of vascular remodeling previously (120). This may be necessary for ECM stabilization in a setting where whole new fibers cannot be constructed. As such, repaired elastin polymers may not achieve full strength as some original peptide bonds cannot be recovered, and total elastin content cannot keep pace with increasing vessel size resulting in thinning of the IEL (32).

### Implications of Extensive Elastic Remodeling During Arteriogenesis

Loss of elastic laminar definition is also a consistent histologic feature of arterial aneurysm development. In some cases, the development of specific types of arterial aneurysms have been linked to flow-mediated remodeling of the IEL, sometimes occurring within collateral arterial networks. For instance, aneurysm degeneration has been observed in mesenteric arterial collaterals in response to isolated celiac occlusion [resulting in pancreaticoduodenal arterial aneurysms (121–123)] or concurrent celiac and superior mesenteric arterial occlusion [resulting in inferior mesenteric arterial aneurysms (124, 125)]. Disruption in the IEL and changes in the media are essential features in the development and propagation of human cerebral aneurysms (126–128). Morphologic assessments of the aneurysm wall have demonstrated malalignment in medial smooth muscle cells and accumulation of macrophages, MMP-9, and myeloperoxidase, essential components of elastin and IEL degradation (129, 130). Degeneration of the IEL and longitudinal elongation (conversion from a contractile to a “synthetic” phenotype) is similarly found at sites of intracerebral aneurysms as well (131).

In the case of cerebral aneurysm pathogenesis, focally increased fluid shear stress provides the local impetus for IEL degradation and enlargement of fenestrations (132–134). It is possible that this would mechanically weaken the vessel wall and predispose to aneurysmal degeneration (132). Notably, cerebral aneurysms are frequently associated with elevated shear stress, and occur more frequently in association with a carotid artery occlusion (130, 135–137). Given the irreplaceable nature of elastic fibers, aggressive diameter expansion risks exhausting local baseline elastin content which can create weakened and aneurysm prone collaterals. The underlying pathology of what may otherwise appear to be disparate manifestations of aneurysmal disease may relate to mechanisms of remodeling like those of arteriogenesis.

### Limitations of Experimental Arteriogenesis Research

The use of arterial ligation in animal models of arteriogenesis typically creates an acute ischemia and is an important limitation regarding extrapolating animal models to human disease which tends to develop chronically. Numerous studies in larger animals, however, have employed surgically placed ameroid constrictors as a method to simulate more gradual arterial occlusion. Human arterial occlusive disease is variable, with (perhaps most commonly) a slowly worsening stenosis in the case of chronic atherosclerosis, but also via sudden arterial occlusion with thromboembolism or *in situ* thrombosis, or even arterial transection in trauma. Evidence of collateral artery formation may be found in all instances.

### Future Therapies

Interventions are currently being directed toward improving arteriogenesis as well as angiogenesis and are under clinical investigation with the hope that these will lead to more effective and non-surgical therapies for AOD. Unfortunately, however, current knowledge of arteriogenesis is limited, and methods to enhance the inflammation and positive remodeling of collateral arteries through growth factor or cytokine supplementation are known to have opposing effects by exacerbating atherosclerosis (138). Developing effective therapies to augment arteriogenesis yet not promote atherogenesis requires more detailed understanding of the molecular mechanisms involved.

## Conclusion

**Figure 3:**
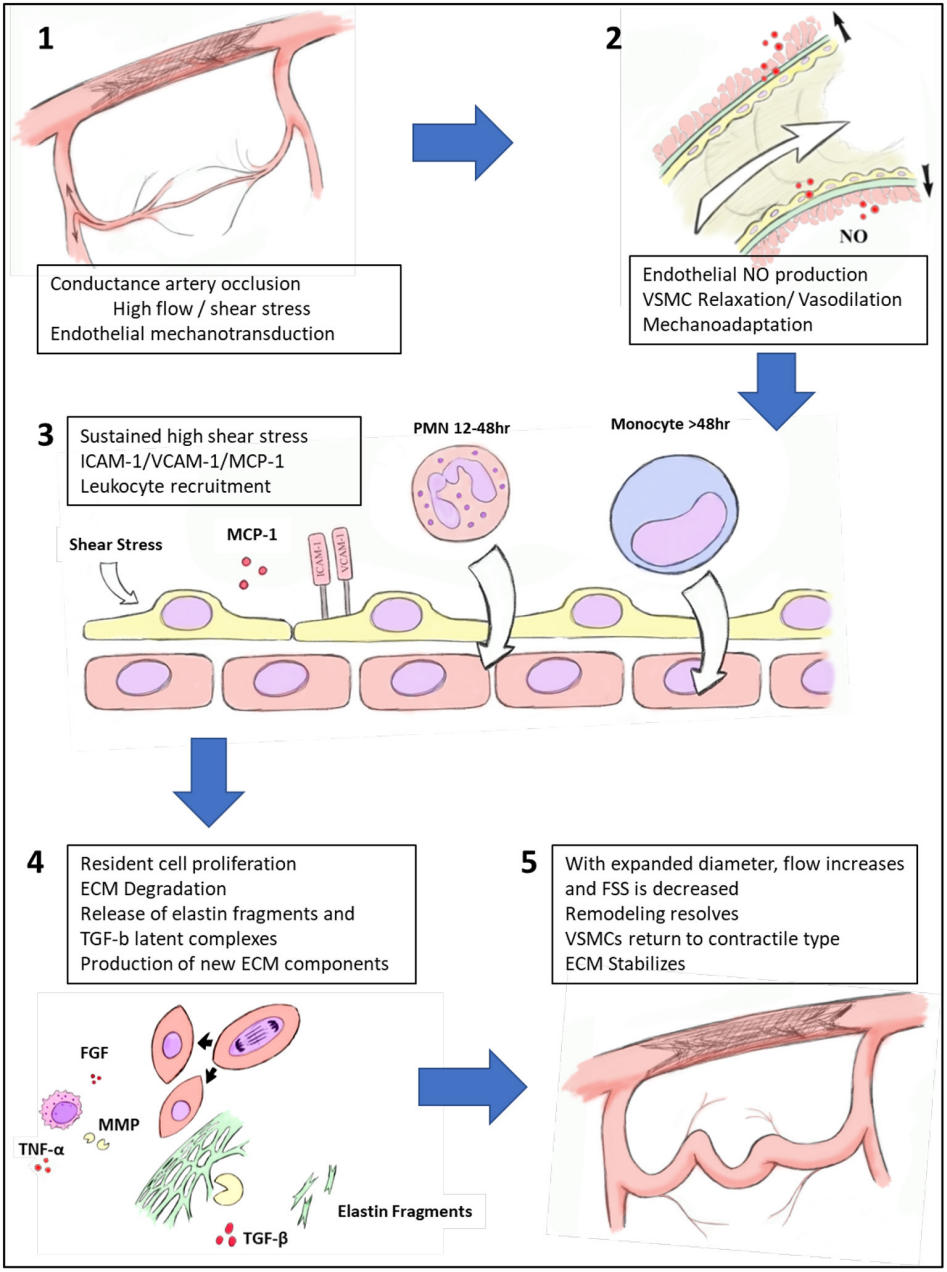
Process of collateral artery recruitment and remodeling. Following occlusion of a conductance vessel, there is immediate increase in flow across pre-existing collaterals. The resulting elevated fluid shear stress (FSS) is recognized by endothelial cells via mechanoreceptors, which leads to nitric oxide (NO) production and subsequent relaxation of vascular smooth muscle cells (VSMCs) and then vasodilation. Prolonged vasodilation produces mechanoadaptation of collaterals. With sustained elevation of FSS, endothelial cells are activated to express intercellular adhesion molecule 1 (ICAM-1), vascular cell adhesion molecule 1 (VCAM-1) and monocyte chemoattractant protein-1 (MCP1) which recruit inflammatory cells to the developing collateral artery. With the aid of a population of perivascular macrophages, growth factors and cytokines such as tumor necrosis factor-alpha (TNF-α), fibroblast growth factor (FGF) are produced, increasing phenotypic modification of VSMCs and proliferation. Elastolytic enzymes such as matrix metalloproteinases (MMPs) are produced, which partially degrade the elastic framework, releasing latent transforming growth factor-beta (TGF-β) complexes. As diameter expands, FSS decreases and the pressure for outward remodeling dissipates. VSMCs return to differentiated phenotype and collateral artery extracellular matrix (ECM) stabilizes.

Arteriogenesis is a complex mechanism for collateral arterial pathways to develop quickly into larger and higher capacity vessels capable of effectively perfusing tissues distal to a conductance vessel occlusion (Figure 3). Increased fluid shear stress, typically caused by flow adaptations in AOD due to large vessel occlusion, initiates endothelial activation ultimately resulting in a cascade of inflammation, cellular proliferation, migration, and tissue remodeling. While details of the molecular signaling processes underpinning arteriogenesis are continually emerging, effective methods to improve collateral development as a clinically useful therapy remain elusive. Continued efforts aimed at manipulating and enhancing functional collaterals promises to reveal possible therapies to medically revascularize patients suffering from practically any manifestation of AOD. However, caution will likely be necessary once adapting to clinical use, as there are shared pathways between arteriogenesis and atherogenesis, and outward remodeling of arteriogenesis may produce weakened ECM structures that theoretically would be at risk for aneurysmal degeneration. Further investigations are required into this field to fully appreciate the molecular cascades involved in linking these processes and constitute potential avenues for continued investigation.

## Author Contributions

Conceptualization and design of the study were performed by RM and RK. Original draft written by RK. RM and EA wrote sections of the manuscript. All authors contributed to the article and approved the submitted version.

## Funding

This work was supported by a grant from the Department of Veterans Affairs (IK-2BX003509) and in part by the Vascular Cures Foundation.

## Conflict of Interest

The authors declare that the research was conducted in the absence of any commercial or financial relationships that could be construed as a potential conflict of interest.

## Publisher's Note

All claims expressed in this article are solely those of the authors and do not necessarily represent those of their affiliated organizations, or those of the publisher, the editors and the reviewers. Any product that may be evaluated in this article, or claim that may be made by its manufacturer, is not guaranteed or endorsed by the publisher.
